# Care of Spontaneous Evolution

**Published:** 1852-09

**Authors:** F. K. Bailey

**Affiliations:** Almont, Michigan


ART. III.—*Case of Spontaneous Evolution.* By F. K. Bailey, M.D.

On the 20th January, 1843, I was called to visit Mrs. D——, aged about 40, and the mother of several children. Found that she had been in labor several hours, and had deferred sending for medical aid, "in hopes to get along without." On examination, found that the left hand and arm had passed so low, that the shoulder could easily be felt. The os uteri was dilated to a considerable extent, and each contraction pressed so hard as to push the hand almost without the os externum.

The whole extremity was so swollen as to fill the vagina, rendering it impossible to pass my hand into the uterus, in order to bring down the feet.

The woman was considerably exhausted, and very much agitated withal. I immediately gave her a full dose of opium, and during each pain pressed as hard as was practicable against the arm. Proceeding in this manner during a dozen or more pains, the shoulder began to recede a little, carrying the arm higher up in the vagina. Encouraged by such appearances, I persevered for nearly half an hour more, when to my joy the arm and hand suddenly went beyond reach. In a short time the feet presented, and the labor was soon finished. The child appeared as though it had been dead some time; it was full grown, weighing over seven pounds.

Cases like the above are rare, this being the only one that has occurred in this region to my knowledge. The rationale of the result in this case may be stated as follows: Each pain tended to push the shoulder into the vagina; after it had advanced to a certain limit no further progress would be made; pressure upward upon the shoulder prevented any advancement, and the contraction of the fundus would push the feet down, and also give a rotary motion to the whole body. After the body of the child had turned to a certain extent, the presenting shoulder and arm would be drawn back into the womb. The action of the fundus was the same as that of one hand of the obstetrician pulling down the feet, while the other pushed upward against the presenting shoulder.

Almont, Michigan, August, 1852.

